# Learning clinical reasoning: how virtual patient case format and prior knowledge interact

**DOI:** 10.1186/s12909-020-1987-y

**Published:** 2020-03-14

**Authors:** Jan Kiesewetter, Michael Sailer, Valentina M. Jung, Regina Schönberger, Elisabeth Bauer, Jan M. Zottmann, Inga Hege, Hanna Zimmermann, Frank Fischer, Martin R. Fischer

**Affiliations:** 1 Institute for Medical Education, University Hospital, LMU Munich, Munich, Germany; 2 Education and Educational Psychology, LMU Munich, Munich, Germany; 3 Medical School, University Augsburg, Augsburg, Germany; 4 Department of Radiology, University Hospital, LMU Munich, Munich, Germany

**Keywords:** Instructional materials/methods, Clinical reasoning, Virtual patients, Case formats

## Abstract

**Introduction:**

Clinical reasoning has been fostered with varying case formats including the use of virtual patients. Existing literature points to different conclusions regarding which format is most beneficial for learners with diverse levels of prior knowledge. We designed our study to better understand which case format affects clinical reasoning outcomes and cognitive load, dependent on medical students’ prior knowledge.

**Methods:**

Overall, 142 medical students (3 rd to 6 th year) were randomly assigned to either a whole case or serial cue case format. Participants worked on eight virtual patients in their respective case format. Outcomes included diagnostic accuracy, knowledge, and cognitive load.

**Results:**

We found no effect of case format on strategic knowledge scores pre- vs post-test (whole case learning gain = 3, 95% CI. -.01 to .01, serial cue learning gain = 3, 95% CI. -.06 to .00 *p* = .50). In both case formats, students with high baseline knowledge (determined by median split on the pre-test in conceptual knowledge) benefitted from learning with virtual patients (learning gain in strategic knowledge = 5, 95% CI .03 to .09, *p* = .01) while students with low prior knowledge did not (learning gain = 0, 95%CI −.02 to .02). We found no difference in diagnostic accuracy between experimental conditions (difference = .44, 95% CI −.96 to .08, *p* = .22), but diagnostic accuracy was higher for students with high prior knowledge compared to those with low prior knowledge (difference = .8, 95% CI 0.31 to 1.35, *p* < .01). Students with low prior knowledge experienced higher extraneous cognitive load than students with high prior knowledge (multiple measurements, *p* < .01).

**Conclusions:**

The whole case and serial cue case formats alone did not affect students’ knowledge gain or diagnostic accuracy. Students with lower knowledge experienced increased cognitive load and appear to have learned less from their interaction with virtual patients. Cognitive load should be taken into account when attempting to help students learn clinical reasoning with virtual patients, especially for students with lower knowledge.

## Introduction

Clinical reasoning has been fostered with varying case formats including the use of virtual patients (VPs; 1). Generally, VPs stimulate clinical reasoning, potentially building diagnostic competencies in medical students [1, 2]. In our study, we utilized VPs and compared two case formats (whole case or serial cue format) with respect to their potential to foster clinical reasoning for students with different levels of prior knowledge. We will first provide an overview of the scientific literature regarding learning with VPs in different case formats. Then we will provide background on the role prior knowledge plays along with its interaction with cognitive load in clinical reasoning.

In VP environments, learners act in simulated scenarios. In these scenarios, learners emulate the role of healthcare providers to obtain a history, and make further diagnostic and therapeutic decisions [1, 3]. To characterize VPs, Huwendiek et al. [4] developed a VP typology which includes instructional design as one of the five main categories. The typology also includes the interactivity type (i.e. the number of cognitive interactions). Similarly, Cook et al. identified “interactivity” and clinical information requests and response" as feature variations in VPs [5]. In VP learning environments, learners typically follow a path through a VP case and access information in a stepwise manner (serial cue format) triggered by interactions (e.g. clicking on navigation buttons or answering questions) with the system. However, when considering the interactions described by Huwendiek [4] and Cook [5], a special case format occurrence provides all information to the learners at once, thus requiring little to no interaction other than entering the diagnosis (whole case format). Such format differences across the VP taxonomy have the potential to induce very different learning processes and patterns of cognitive load. However, it has not yet been investigated how cognitive interactions may influence success in learning clinical reasoning processes.

There are two knowledge facets of diagnostic competencies to be distinguished: conceptual and strategic knowledge [6]. When conceptual knowledge is organized around diseases, it is known as illness scripts. Oftentimes this is used as prior knowledge in clinical reasoning studies. Strategic knowledge comprises clinical problem-solving strategies and heuristics [6]. The serial cue format simulates a problem-solving approach that approximates everyday experiences of clinicians and thus has an intuitive appeal to medical educators [7]. Schmidt and Mamede conclude that the serial cue design might be more beneficial for advanced students with high prior knowledge because they have already developed functional illness scripts, enabling them to navigate through the case, searching for new information without experiencing excessive cognitive load [7]. However, it is also plausible that the whole case design is more beneficial for students early in their learning. The rationale is that they are freed from the challenge of determining what information should be collected, allowing greater capacity to internalize the knowledge to be learned. The whole case format can be considered a specific form of worked examples, which are known to help novice students [8]. In fact, Nendaz et al. [9] showed that diagnostic accuracy was higher for the whole case format than for the serial cue format when presented to students, residents, and practitioners in a paper-based comparative study.

Intrinsic cognitive load is the inherent level of difficulty associated with a specific problem-solving task [10]. As a result, students with low prior knowledge are expected to experience higher intrinsic cognitive load than high prior knowledge students [10]. Features of digital learning environments that are not related to the problem-solving task itself (e.g., background noise; additional and unnecessary information in and functionality of the digital environment) can produce additional load, known as extraneous cognitive load. If intrinsic and extraneous cognitive load are high, they may lead to cognitive overload, thus decreasing or entirely impeding learning. It is generally recommended to reduce extraneous cognitive load in order to free cognitive capacity for learning processes [10, 11].

Pollock et al. [12] showed that presenting reduced information in a first learning phase and full information in a second learning phase resulted in lower cognitive load and better learning compared to full information being presented in both phases. However, empirical evidence of these claims in medical education is sparse. In order to better understand students’ learning processes, it has been suggested that cognitive load should be investigated in health professional education and research on clinical reasoning [11, 13].

The gap in the literature we address is an empirical one. In their review Schmidt and Mamede [7] conclude that serial cue might be better for advanced students, but could not include an experimental study directly investigating this comparison for students with different levels of prior knowledge. Further, it is deemed necessary to control for cognitive load in VP learning environments, yet the actual evidence in relation to clinical reasoning outcomes sparse. We designed our study to directly fill this gap and investigate how case format and medical students’ prior conceptual knowledge affect the clinical reasoning outcomes (strategic knowledge, diagnostic accuracy), and cognitive load.

We addressed the following research questions (RQ):

- RQ1: What are the effects of VP case format (serial cue versus whole case) and prior knowledge (low versus high) on strategic knowledge and diagnostic accuracy?
- Hypothesis 1: Students with high prior knowledge will gain more in strategic knowledge and a higher diagnostic accuracy compared to students with low prior knowledge, independent of the case format.
- Hypothesis 2: There will be an interaction between case format and prior knowledge with the serial cue format being more effective in facilitating strategic knowledge and diagnostic accuracy for students with high prior knowledge and the whole case format being more effective in facilitating strategic knowledge and diagnostic accuracy in students with low prior knowledge.
- RQ2: To what extent does the case format generate cognitive load in students with different levels of prior knowledge?
- Hypothesis 3: Students with low prior knowledge will experience higher intrinsic cognitive load when working on VPs, independent of the format.
- Hypothesis 4: Students with low prior knowledge will experience higher intrinsic cognitive load in the serial cue than in the whole case format.

With testing these hypotheses we intend to generate evidence for the best practice in teaching clinical reasoning and fostering strategic knowledge for any level of prior student knowledge without overwhelming their cognitive abilities.

## Method

### Design and sample

The study employed a 2 × 2 design with case format and prior knowledge as factors. The total sample consisted of *N* = 142 medical students (mean age = 24.4; SD = 2.9; 72% female), all from above the third year of a six-year curriculum. Students were randomized to case format condition, which contrasted the whole case format (*N* = 71) and the serial cue format (N = 71) in a between-subjects design. Participants were divided into low and high prior knowledge using a median split based on the results from a conceptual knowledge pretest: The low knowledge group (*N* = 59) scored 0–4 points on that test and the high knowledge group (*N* = 83) scored 5–10 points. We sought an overall sample size of at least *N* = 120 based on a-priori power analyses intended to reveal effects with a power of .90 for medium-sized effects (partial eta^2^ = .08).

### Learning environment

Participants worked on eight VPs in the learning environment CASUS (http://www.casus.net). Learners were asked to adopt the role of a general practitioner and to diagnose eight VPs without time restrictions. Learners were given all the relevant and some irrelevant diagnostic information and could submit a diagnosis whenever they felt ready. Four of the VPs concerned patients with the key finding of back-pain; the other four concerned patients with fever as the leading symptom. The VPs were selected in order to have students apply clinical reasoning skills. We did not tell the students the subject areas to avoid bias. The learning environment tracked the time students spent working on the VPs.

### Case formats

#### Whole case format

All clinical information for the VP, including media elements, was presented in one long text. The final diagnosis was submitted through clicking a button on the bottom of the page.

#### Serial cue format

**Fig. 1:**
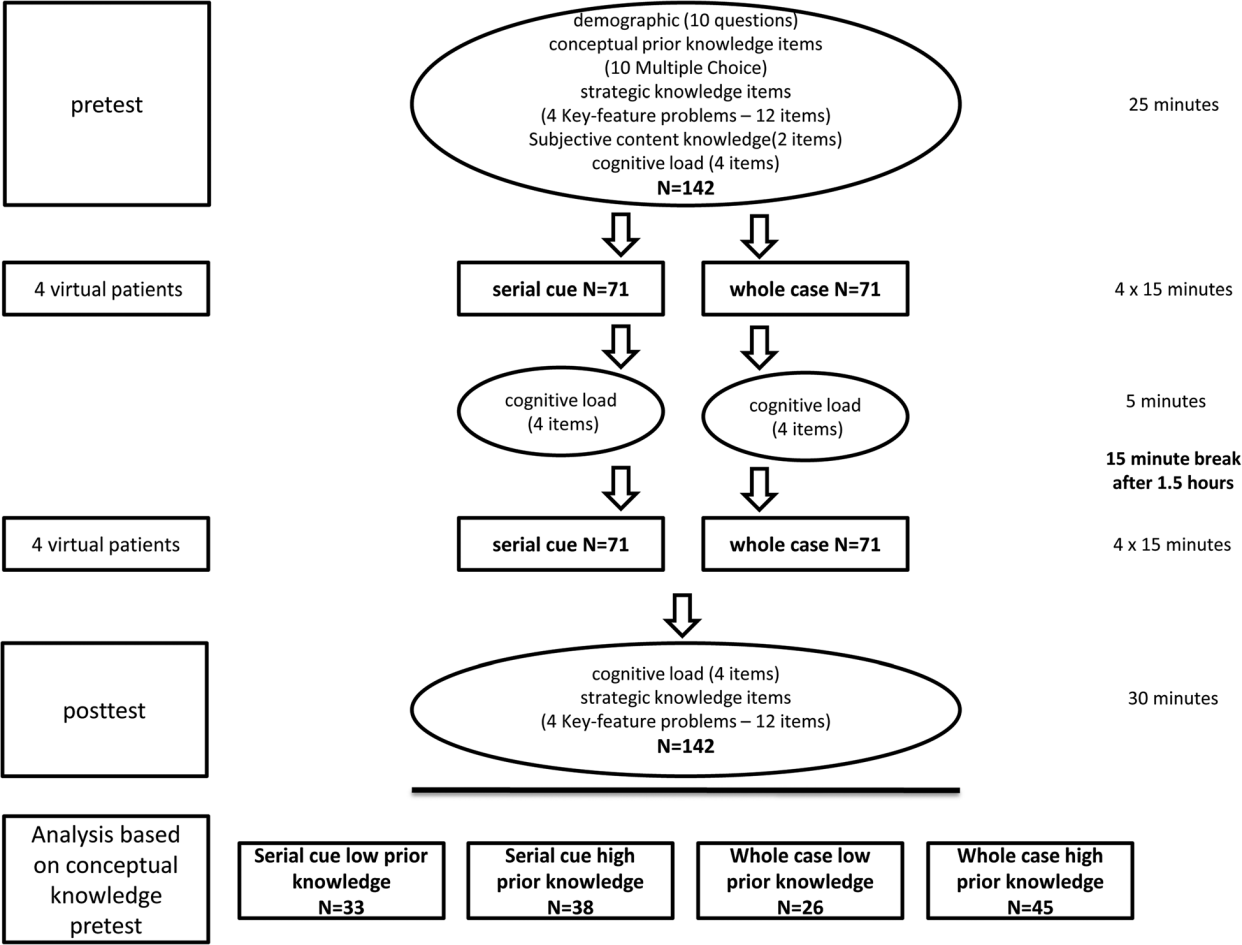
Procedure of our study

The same information for the VP was presented on different pages, called cards, with each card referencing one test, examination, and laboratory result (see lower part of Fig. 1). After the initial information of the VP was presented, learners could choose from a menu and decide which of the tests or examinations they wanted to see next. After choosing one test/examination and having seen the results, learners could decide to choose another or to enter their final diagnosis.

No feedback was provided during the study. After the students were finished with the study they were provided with the correct final diagnoses on demand. All cases were written, reviewed and revised in three rounds with three experienced physicians (one general practitioner, two internists).

### Assessment of conceptual knowledge

To test preexisting conceptual knowledge related to the VP content, a multiple-choice questionnaire with ten items was created. In a pilot test, reliability of the test was Cronbach’s α = .64. Additionally, to the measured conceptual knowledge, we asked for subjective prior content knowledge on a five-point Likert scale (“how much knowledge on the topic fever/back pain do you think you have?”) before the conceptual knowledge test. The subjective content knowledge and performance in the conceptual knowledge test had a small correlation (r = .16).

### Assessment of knowledge and diagnostic accuracy

Strategic knowledge was tested through pre- and posttests consisting of eight key-feature problems with 24 items (three items per problem). This test mirrored the content of the VPs. Scores were reported as percent correct. To avoid a retest effect, the knowledge test was piloted on a sample of the study population and divided in two, creating paired items for knowledge pretest and knowledge posttest based on difficulty. Reliability in the pilot was Cronbach’s α = .62 for the knowledge pretest and α = .63 in the knowledge posttest, respectively. Learning was considered the difference between pre- and posttest strategic knowledge scores.

Additionally, the final diagnosis of each case was determined against the initially developed correct diagnosis. For each correct diagnosis one point was given, and a false diagnosis resulted in zero points. The number of correct diagnoses was used as an indicator of diagnostic accuracy (Range: 0–8 points).

### Cognitive load

Participants’ cognitive load was measured before working on the VPs, after four VPs, and before the posttest. We utilized the 4-item scale constructed by Opfermann [14] for intrinsic (single item: “how difficult do you find the topic at this moment?”) and extraneous cognitive load (three items, e.g. “how difficult is it for you to distinguish important and unimportant information in the learning environment”). The scale was tested in diverse learning environments, with results suggesting a two-dimensional cognitive load (extraneous and intrinsic) rather than a unidimensional scale [14]. We follow Opfermann’s suggestion to embed both dimensions. The scale reliability for extraneous cognitive load was Cronbach’s α = .71.

### Demographic information

Students were asked for their age, gender, year of study, prior grades and prior clinical experiences in a short questionnaire.

### Procedure

Participants were assigned to one testing time slot in a computer lab from mid-October to mid-November of 2017. Upon arrival, participants were given a code that randomly assigned them to either the whole case (*N* = 71) or the serial cue (N = 71) format. Participants filled out the demographic information, self-assessed prior knowledge, completed the conceptual knowledge test and the strategic knowledge pretest, answered cognitive load items and then started working on the VPs in their respective format. After four VPs, participants filled out the cognitive load items a second time. Regardless of their progress, students were given a 15-min break after 1.5 h. Upon completion of the last VP, participants filled out the cognitive load items and the strategic knowledge posttest. Participants received a monetary compensation of 50€ for their participation. The procedure of the study is depicted in Fig. 1.

### Ethical approval

Ethical approval for the study was obtained from the Ethics Committee of the Medical Faculty of LMU Munich (No. 17–249).

### Statistical analyses

Data were analyzed using SPSS 24.0 (IBM Inc.). Sample size and power analysis were performed using G*Power 3 [15]. For gender comparison, a Chi^2^ test was performed. For age comparison, a t-test for independent samples was performed. For all interval scaled data that were measured once (e.g., diagnostic accuracy), an ANOVA was performed. For all interval scaled data that were measured several times (e.g., strategic knowledge pre- and posttest, cognitive load) a repeated measures ANOVA was performed. Alpha level was set to probability *p* < .05. Effect sizes are presented as partial eta^2^ (p.eta^2^) and interpreted according to Cohen [16]. Correlations presented are Pearson correlation coefficients (*r)*.

## Results

### Preliminary analyses

The case format groups did not differ in terms of their age and gender, conceptual knowledge and or strategic knowledge pretest, Post-hoc tests (least significant difference) confirmed that overall cognitive load did not differ between the two groups before they commenced working on the VPs (see Table 1). Conceptual knowledge test scores correlated significantly with semesters studied (*r* = .2, *p* = .04), supporting the construct validity of the test. Time spent working on the VPs (from the first presentation of information to entering the diagnosis), aggregated across all 8 cases, was a mean of M = 45.1 min (SD = 12.2). No differences were found between the case formats (serial cue M = 42.5 min; SD = 9.9 min, whole case M = 41.9 min, SD = 9.3 min, *p* = .80). As well, no difference in time spent was found between those with low and high prior knowledge (M = 42.0 min; SD = 9.4, and M = 42.41; SD = 9.8, respectively, *p* = .85).

**Table 1:** Cognitive Load Scores

VP format	Prior knowledge	Before VPs Mean (SD)	After 4 VPs Mean (SD)	After 8 VPs Mean (SD)
Intrinsic cognitive load				
Serial cue	low	3.31 (.64)	3.38 (.79)	3.44 (.84)
	high	3.11 (.50)	3.00 (74)	3.24 (.71)
	Overall	3.20 (.58)	3.17 (.78)	3.33 (.76)
Whole case	low	3.32 (.56)	3.32 (.85)	3.52 (.83)
	high	3.11 (.65)	2.93 (.81)	3.00 (.80)
	Overall	3.19 (.62)	3.07 (.84)	3.19 (.84)
Overall	low	3.32 (.60)	3.35 (.81)	3.47 (.83)
	high	3.11 (.59)	2.96 (.77)	3.11 (.76)
	Overall	3.19 (.60)	3.12 (.81)	3.26 (.81)
Extraneous cognitive load				
Serial cue	low	2.51 (.75)	2.78 (.88)	2.79 (.92
	high	2.61 (.63)	2.60 (.87)	2.62 (.75)
	Overall	2.57 (.68)	2.68 (.87)	2.70 (.83)
Whole case	low	2.56 (.64)	3.04 (.89)	3.05 (.83)
	high	2.70 (.84)	2.56 (.80)	2.61 (.92)
	Overall	2.65 (.78)	2.72 (.86)	2.77 (.91)
Overall	low	2.53 (.70)	2.89 (.88)	2.91 (.88)
	high	2.66 (.74)	2.57 (.83)	2.62 (.84)
	Overall	2.61 (.73)	2.70 (.86)	2.74 (.87)

In the serial cue format, a mean of 72% of the cues were selected (range: 8–12 cues/case).

### Results regarding RQ1

To identify whether students with high prior knowledge had a higher learning gain in strategic knowledge from pretest to posttest, a repeated measures ANOVA with case format, and prior knowledge was conducted. Results showed statistically significant differences regarding the strategic knowledge gain from pretest to posttest; a difference of 3% (95% CI .01 to .05, *p* = .01, partial eta^2^ = .05) with a small effect size. The analysis further revealed a statistically significant interaction effect of prior knowledge for the knowledge gain (*p* = .01, p.eta^2^ = .06). For low prior knowledge no difference between pretest and posttest was observed (95%CI −.02 to .02) and for high prior knowledge a difference of 5% was observed (95% CI .03 to .09), the latter difference indicating a small to medium effect size. This indicates that students with high prior knowledge had a gain in strategic knowledge and students with low prior knowledge had hardly any knowledge gain (see Table 2).

**Table 2:** Strategic Knowledge Scores (% Correct)

Case format	Prior knowledge	Pretest Mean (SD)	Posttest Mean (SD)
Serial cue	low	0.60 (.07)	0.61 (.11)
	high	0.60 (.08)	0.64 (.08)
	Overall	0.60 (.07)	0.63 (.10)
Whole case	low	0.61 (.06)	0.59 (.08)
	high	0.62 (.08)	0.67 (.11)
	Overall	0.61 (.07)	0.64 (.10)
Overall	low	0.60 (.06)	0.60 (.10)
	high	0.61 (.08)	0.66 (.10)
	Overall	0.60 (.07)	0.63 (.10)

In terms of diagnostic accuracy, students with high prior knowledge performed better diagnosing the VPs compared to those with low prior knowledge (difference of .8; 95% CI 0.31 to 1.35, *p* < .01; p.eta^2^ = .07) with a medium effect size (Low prior knowledge M = 3.97 points; SD = 1.56; high prior knowledge M = 4.81 points; SD = 1.52) Overall, these data support hypothesis 1.

The main effect for case format and the interaction effect for case format and prior knowledge in the ANOVA described above is analyzed to determine the outcome for hypothesis 2. Specifically, whether the two case formats differ in facilitating strategic knowledge and diagnostic accuracy for students with high and low prior knowledge. There was no main effect for case format (whole case difference of 3, 95% CI. -.01 to .01, serial cue difference of 3, 95% CI. -.06 to .00 *p* = .50). There was no interaction effect for case format and prior knowledge (*p =* .49) between pretest and posttest. Diagnostic accuracy between the two case formats did not differ (*p* = .22; serial cue M = 4.23 points; SD = 1.35, whole case M = 4.68 points; SD = 1.77).

Hypothesis 2 did not find support in our empirical data; in summary, the case format did not affect the students’ strategic knowledge and diagnostic accuracy.

### Results regarding RQ2

No statistically significant interaction was found between serial cue and whole case for both cognitive load dimensions (for differences see Table 1, *p* = .24). To determine the cognitive load in the process we compared the differences in extraneous cognitive load for the experimental conditions as well as for low vs. high prior knowledge. Further we compared the differences between before working on the VPs, after having worked on four VPs and after having worked on eight VPs. The interaction effect for the case format and the process was not statistically significant: None of the two case formats does evoke lower extraneous cognitive load (for differences see Table 1, *p* = .99). Hypothesis 3 is not supported by our findings.

The interaction effect of prior knowledge and the process was statistically significant: Students with low prior knowledge experienced higher overall cognitive load compared to high prior knowledge students when working on the VPs (*p* = .43; p.eta^2^ = .05). The increase on overall cognitive load for low prior knowledge students from before working on the VPs to after eight VPs was .38 and significant (*p* < .01, p.eta^2^ = .06) with a medium effect size compared to a non-significant decrease in extraneous cognitive load for high prior knowledge students from before working on the VPs to after eight VPs. The difference in extraneous cognitive load after four and eight VPs between students with low prior knowledge and students with high prior knowledge was statistically significant (difference after 4 VPs of .32, *p* < .01, p.eta^2^ = .06 and difference after eight VPs .29, *p* < .01, p.eta^2^ = .04).

**Fig. 2:**
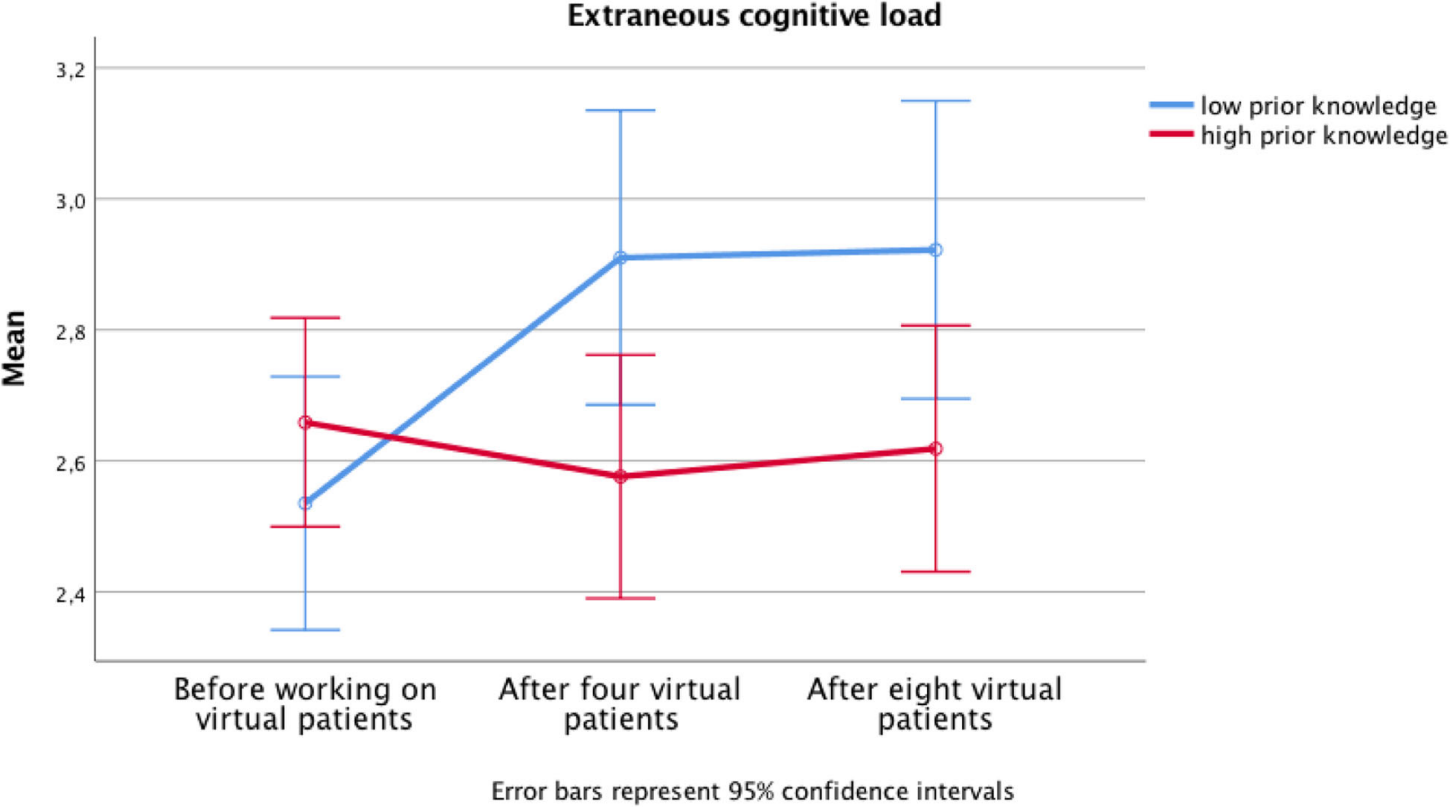
Increase in extraneous cognitive load and difference between the students with low and high prior knowledge scores

Intrinsic cognitive load was significantly higher for students with low prior knowledge at all three times of measurement, compared to high prior knowledge students (difference prior VPs of .22, *p* < .05, p.eta^2^ = .03, difference after four VPs .39, p < .05, p.eta^2^ = .04, difference after eight VPs .36, p < .05, p.eta^2^ = .03). No relevant in- or decrease during the process was observed. Hypothesis 4 is thus supported by our data (see also Fig. 2 and Table 1).

## Discussion

Our study compared the effect of two VP case formats on strategic knowledge and diagnostic accuracy of students with high versus low prior knowledge. Only students with high prior knowledge displayed a substantial benefit in strategic knowledge and diagnostic accuracy, regardless of the case format. This may have occurred because students with lower prior knowledge experienced a higher amount of overall cognitive load. In our as in other studies it has been shown that intrinsic cognitive load is higher in those students with lower prior knowledge [6, 10].

The measured outcomes of learning (strategic knowledge and diagnostic accuracy) did not differ for serial cue vs. whole case presentations of VPs, not even in combination with low vs high prior knowledge students. Further our measurement of prior knowledge relates back to our conceptual knowledge test scores, which only assessed two very specific knowledge aspects and not overall prior knowledge. Yet the scores correlated significantly with semesters studied supporting the construct validity of the test. Extraneous and intrinsic cognitive load were higher for learners with low prior knowledge. This was to be expected for intrinsic cognitive load [6, 10] and is somewhat surprising for extraneous cognitive load. However, it might be the case that the items as we used them in our study asked whether learners had a hard time to distinguish relevant and irrelevant information. This might be highly relatable to the construct of intrinsic cognitive load. The task-load in itself is often lower in learners with high prior knowledge [6, 10].

However, the learning environment with VPs without further guidance might have evoked extraneous cognitive load so high that gains in strategic knowledge might not have been possible for students with low prior knowledge. Learning environments with extraneous cognitive load low enough to allow for learning seem to be necessary to achieve progress in clinical reasoning for students with low prior knowledge, independent of the case format. There is still limited knowledge on how factors innate to the learner, their abilities, prior knowledge, and factors of the learning environment, like the case format, design and VP interactivity influence the learning of clinical reasoning. Our results indicate the need to carefully consider not only which case format suits students best, but to determine which subject-specific prior knowledge students should possess in order to be able to learn with VPs. Interestingly, we didn’t find any difference in the time the students spent working on the cases, neither for the case formats, nor for the prior knowledge. It could be that very experienced students in a field might be faster in finding the relevant information in the serial cue case format.

An earlier study found that the diagnostic accuracy for students through the presentation of the cases in the whole case format was higher compared to the serial cue format [9]. Our results, however, show no difference due to the format, neither in the strategic knowledge gain of students nor in the diagnostic accuracy of the VPs. The differing result might be due to several aspects where the two studies vary. In the earlier study [9], two paper-based patient cases with direct communication with an investigator on a study sample of students, residents and general internists were applied. It is possible that their students of low prior knowledge experienced high intrinsic cognitive load because the paper-based cases were not designed for students and were too difficult. It is also possible that the necessity to communicate with the facilitator in the serial cue format was too demanding. For future research, the implementation of procedural measurements, such as cognitive load, is especially important considering that emerging paradigms of a more social learning of clinical reasoning [17, 18] require students to manage more adaptive and interactive objects on their screen, compared to individual learning. This may result in additional extraneous cognitive load. Despite the growing number of design guides for VPs [1, 5, 6], there still is a research gap regarding how medical educators can best implement VPs in a way that students are not cognitively overwhelmed by the learning environment, especially those with low prior knowledge.

### Limitations

Our study has several limitations. First, we did not utilize any representation, reflection, feedback, or self-explanation prompts found to be helpful for fostering clinical reasoning in students. It is possible that differences in clinical reasoning outcomes between the case formats only become apparent when combined with scaffolding and feedback. Further studies should compare VP case formats with additional scaffolding.

**Fig. 3:**
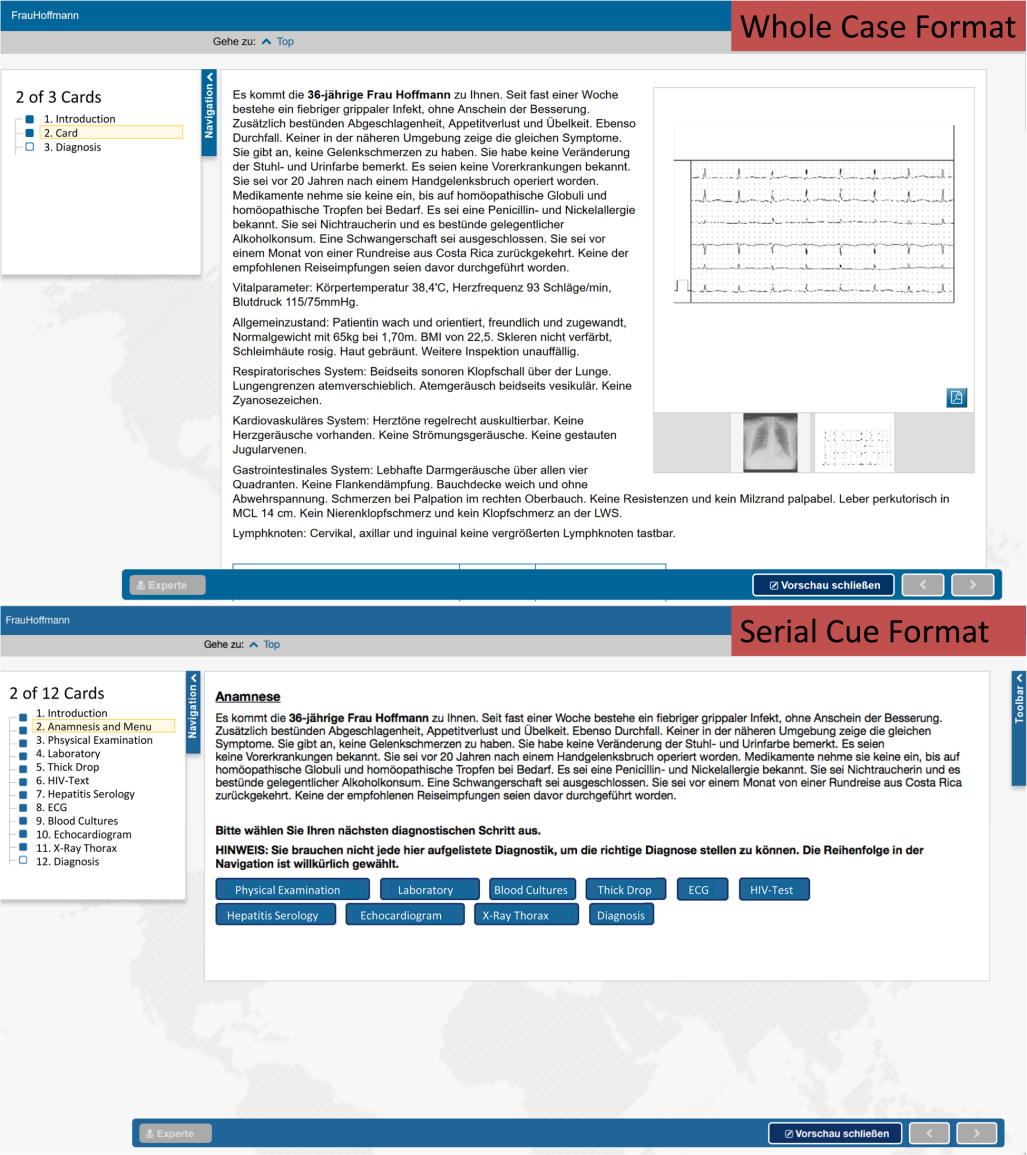
Screenshots of the two case formats for the virtual patients: On the upper part represents the whole case format, with all information written on one card. On the lower part the serial cue format is presented, with buttons to redirect to the different cards

Second, the specific design of the case formats might have been too similar to find effects. For example, the whole case format included pictures that needed to be enlarged for interpretation (see Fig. 3), similarly to a serial cue format. There are also VPs that incorporate a much larger number of cues, such as Web-SP. [19] Nonetheless, we think that the case formats are implemented quite stereotypically. However, whether the whole case, as a non-interactive VP can still be defined as a special VP occurrence is questionable. In our manuscript we used the term VP in the broadest sense for the whole case as well as for the serial cue case format. Further studies could incorporate a larger number of cues in order to see whether this affects the findings in relation to prior knowledge.

Third, we cannot completely rule out that innate learning abilities or subject specific prior experiences of students might be overrepresented in one of the groups. Further, our findings might be subject-specific for our VP content. However, we did randomize students to the groups and assess the subject specific knowledge which should restrict the risk of overrepresentation. Findings need to be replicated with other VP content.

Fourth, the overall cognitive load was high for both case formats among students with low prior knowledge. This may have prevented us from finding effects of the case format. Familiarizing learners with very easy content in the learning environment or demonstrating each step in a guided session could potentially reduce extraneous cognitive load over both case formats.

Fifth, the pilot test for the knowledge pre- and posttest revealed a Cronbach’s alpha of .62 and .63, which is not ideal, while .7 is considered good [16]. However the assessments are still reliable enough to allow for between-group comparisons and the study sample was deemed sufficiently powered with 1-β = .80 by a post-hoc power analysis with G*power (www.gpower.hhu.de).

## Conclusions

Advanced learners can improve their clinical reasoning when working on VPs. Before including less advanced learners medical educators should consider how to ensure that learners profit from VPs. One way to make VPs profitable for less advanced students could be as reflection prompts with instructional support adapted to the prior knowledge of learners [20] or even to incorporate other instructional methods before working on the VPs. While parts of the design for VPs are important to foster clinical reasoning, we found that testing students’ actual subject-specific prior knowledge levels is also of great value. Measurement of cognitive load is necessary to identify under which conditions learning with VPs can be optimized or hindered. Further research should consider instructional interventions such as adaptive feedback [21] and reflection [22], self-explanation [23], and representation prompts [24], which might influence the learning of clinical reasoning by potentially lowering extraneous cognitive load and increasing knowledge gains for learners.

## Data Availability

Raw data is available from Jan Kiesewetter on request.

## Abbreviations

RQ: Research Question
VP: Virtual Patient
VPs: Virtual Patients
